# Practical Comprehensive Approach to Current Atrial Fibrillation Challenges: Insights from an Expert Panel

**DOI:** 10.3390/jcm14155199

**Published:** 2025-07-22

**Authors:** Carlos Escobar, Miguel Camafort, Elena Fortuny, Maxim Grymonprez, Alejandro Isidoro Pérez-Cabeza, Tine L. de Backer, Leaders Connect Group

**Affiliations:** 1 Cardiology Service, University Hospital La Paz, 28046 Madrid, Spain; 2 Internal Medicine Service, Atrial Fibrillation Unit, Hospital Clínic Barcelona, 08036 Barcelona, Spain; camafort@clinic.cat; 3 Fundació de Recerca Clínic Barcelona-Institut d’Investigacions Biomèdiques August Pi i Sunyer (IDIBAPS), 08036 Barcelona, Spain; 4 Centro de Investigación Biomédica en Red Fisiopatología de la Obesidad y Nutrición (CIBER-OBN), 15706 Santiago de Compostela, Spain; 5 Cardiology Service, The Health Research Institute of the Balearic Islands (IdISBa), University Hospital Son Espases, 07120 Palma de Mallorca, Spain; elenafortunyfrau@gmail.com; 6 Pharmaceutical Care Unit, Department of Bioanalysis, Faculty of Pharmaceutical Sciences, Ghent University, 9000 Ghent, Belgium; 7 Cardiology Service, Clinic University Hospital Virgen de la Victoria, 29590 Málaga, Spain; alejandroipcabeza2@gmail.com; 8 Centro de Investigación en Red de Enfermedades Cardiovasculares (CIBERCV), 28029 Madrid, Spain; 9 Internal Medicine and Pediatrics, Department of Cardiology, Ghent University Hospital, 9000 Ghent, Belgium; tine.debacker@ugent.be

**Keywords:** adherence, atrial fibrillation, catheter ablation, comorbidity, hemorrhage, oral anticoagulation, thromboembolic risk, ageing

## Abstract

**Background/Objectives**: Atrial fibrillation (AF) is a very common arrhythmia and the main cause of embolic events. Early diagnosis and treatment are crucial to prevent thromboembolic events. Although DOACs are an important advance in AF management, optimization is required. This study aims to evaluate the newly available evidence and experts’ opinions on the clinical care of AF patients and to develop a set of practical recommendations to improve the management of patients with AF. **Methods**: A questionnaire was developed on the topics of AF diagnosis, stroke prevention, rate and rhythm control, and management of comorbidities, based on the scientific committee’s judgment and a rapid literature review. The level of agreement of the panelists with each statement was evaluated using the Likert 5-point scale. The results of the questionnaire were discussed in a final meeting and practical recommendations were made. **Results**: Thirty-five Spanish panelists, all experts in AF management, answered the questionnaire. Most of the statements (78%) reached the levels of agreement or unanimity. Discrepancy (9%) and rejection (13%) were also reported. **Conclusions**: This study underscores the importance of a 12-lead electrocardiogram to diagnose AF, with wearable devices serving as useful tools; catheter ablation as a superior strategy for restoring and maintaining sinus rhythm compared to pharmacotherapy; the importance of comorbidity management to reduce incidence and recurrence of AF; adherence and persistence as critical factors for the efficacy and safety of anticoagulation; and the preference for DOACs, particularly apixaban and edoxaban, for stroke prevention in patients ≥75 years old or with chronic kidney disease.

## 1. Introduction

Atrial fibrillation (AF) is the most common arrhythmia in Western countries and is associated with high morbidity and mortality. It is estimated that 4–5% of adults older than 40 years suffer from AF, with a notably increasing trend in people aged ≥65, where it exceeds 10% [1,2,3,4]. The estimated worldwide prevalence was 50 million in 2020 [5]. Atrial fibrillation is the main cause of embolic events and is associated with heart failure and cognitive and quality of life impairment [2], representing a substantial healthcare and economic burden for health systems and patients [6].

The mainstay treatment for preventing thromboembolic events in patients with AF consists of oral anticoagulants (OACs) [7], and currently, direct oral anticoagulants (DOACs) are preferred over vitamin K antagonists (VKAs). For decades, VKAs were the only option, despite their slow onset, numerous interactions with food and other drugs, and the requirement of regular monitoring of coagulation [7]. In recent years, DOACs, which include apixaban, edoxaban, rivaroxaban, and dabigatran, have become the treatment of choice for the prevention of stroke and systemic embolism in patients with non-valvular AF, as recommended by the main international clinical practice guidelines [7,8].

Although the introduction of DOACs has led to improved prevention of stroke and peripheral embolism [9], further progress is still required to optimize the management of patients with AF. Relevant evidence has emerged in several areas [9], including diagnoses facilitated by wearable devices, the importance of early intervention to maintain sinus rhythm, research aimed at optimizing the selection of patients for ablation, as well as publications on new ablation techniques. Growing evidence also highlights the issues arising from poor adherence and persistence to anticoagulant therapy, with a reduction in treatment effectiveness and a worsened prognosis being particularly concerning [10]. In a recent study conducted in Belgium [11], it was observed that adherence and persistence to DOAC treatment is higher compared to VKAs, with edoxaban showing better persistence rates compared to apixaban, dabigatran, and rivaroxaban. Additionally, once-daily DOAC regimens had higher adherence than twice-daily schedules [11].

Given these challenges, optimizing AF management requires improving diagnostic strategies and leveraging opportunities provided by emerging technologies. Additionally, the effective management of comorbidities and associated risk factors—central to the care of AF patients, as emphasized in the AF-CARE algorithm proposed by the 2024 ESC guidelines [7]—remains a priority. Further advancements are also needed in enhancing anticoagulation strategies, improving patient adherence and persistence to treatment, and addressing unresolved issues in rhythm control for patients with AF.

Currently, important gaps in knowledge remain that cannot be fully addressed with the existing evidence from available clinical trials. In this context, the expert consensus may help to standardize and support decision-making in the management of AF patients. Therefore, this study aims to evaluate newly available evidence alongside experts’ opinions concerning clinical care of AF patients and to develop a series of practical recommendations to optimize the management of patients with AF.

## 2. Materials and Methods

This document presents the findings of a rapid literature review followed by a single round of a questionnaire to a panel of experts in AF, followed by an in-person discussion of the results of the questionnaire. Before conducting the literature review, the key areas and gaps in the management of AF to be addressed were defined by a scientific committee. As a result, four distinct topics were covered, corresponding to: (i) AF diagnosis, (ii) stroke prevention, (iii) rate and rhythm control, and (iv) management of comorbidities.

The scientific committee comprised six experts in the management of patients with AF and specialists in cardiology or internal medicine. One of the experts acted as the project coordinator (CE). Four of the members were from different areas in Spain, while two were from Belgium.

A research protocol was developed to describe the methodology of the project, which was validated by the scientific committee, and 19 clinical questions were formulated.

The funder had no role in the development of the questionnaire, the expert discussions, or the formulation of the practical recommendations. The funder was involved only in the selection of panelists, based on predefined criteria that included expertise in the management of atrial fibrillation, scientific publications in the field, and geographical representation.

Literature review

To conduct the literature review, a search strategy protocol, which included the main inclusion and exclusion criteria of the different publications, was developed and can be found in the Supplementary Materials (Table S1).

Publications related to AF from the databases PubMed and Epistemonikos were included. A stepwise search was conducted, the first round of which involved assessment of the publications according to inclusion criteria by title and abstract. In a second round, the full texts were read. The scientific committee considered additional publications for inclusion if they were relevant and were not on the initial list. The final publications included in the literature review were selected according to topic relevance and expert judgment. Finally, a total of 32 publications were included in the synthesis of evidence. The list of the included publications is shown in Table S2.

Questionnaire development

The scientific committee developed the questionnaire, which comprised 32 statements, based on the four proposed topics. The level of consensus was assessed using the Likert 5-point scale [12] and the following response range: ‘strongly disagree’, ‘disagree’, ‘neutral’, ‘agree’, or ’strongly agree’.

A panel of 35 experts in AF from various hospitals and geographic areas in Spain was selected and invited to respond to the questionnaire. They had to meet the following criteria: (i) specialist in cardiology or internal medicine; (ii) proven experience in the management of patients with AF; (iii) consultation with a reasonable number of AF patients per month; and (iv) availability to respond to the questionnaire.

The questionnaire was available online for four weeks, and the panelists were invited to leave comments to justify or clarify their responses.

In the interpretation of results, agreement with a statement was defined as ≥80% of panelists voting ‘agree’ or ‘strongly agree’. Unanimity was defined as a 100% level of agreement. Discrepancy was defined as an agreement level between 66% and 79%, and rejection as an agreement level below 66%.

Discussion meeting and formulation of practical recommendations

Results were analyzed, and a final in-person meeting with all the members of the scientific committee and the panelists was carried out. The aim of this meeting was to discuss the main results and define the conclusions of this study and come up with practical recommendations. The discussion was guided by the scientific committee, and the panelists were divided into two groups to show the results of the questionnaire and facilitate the debate. For the construction of practical recommendations, four groups corresponding to the different thematic blocks of the questionnaire were formed. Recommendations, based on the results of the questionnaire, were then proposed and discussed by the entire expert team until agreement was reached.

## 3. Results

### 3.1. Participants

The questionnaire was answered by 35 cardiologists or internal medicine physicians experienced in AF management. Table 1 presents the distribution of panelists based on their medical specialty, the type of institution (hospital or outpatient clinic), their membership in a working group of a scientific society, their geographical localization within Spain, and their clinical experience.

The results indicate that most participating panelists were cardiologists (94%) practicing in hospital settings (94%). Sixty-six percent of participants belonged to a working group of a scientific society. All participants worked with at least 25 patients per month who had AF and were treated with any OAC, and 94% of participants had more than 10 years of experience in AF management. Their geographical distribution was broad, representing 14 out of the 17 autonomous regions of Spain.

### 3.2. Questionnaire Results

Table S3 shows the statements of the questionnaire, grouped into four different AF sub-topics (diagnosis, stroke prevention, rate–rhythm control, and comorbidities), and the level of agreement for each one.

Panelists agreed with most of the statements on the questionnaire (78%). Within these statements, 59% were defined as agreement and 19% unanimity. Nine percent of the statements were defined as discrepancy, while 13% of the statements were rejected.

### 3.3. Practical Recommendations

**Figure 1:**
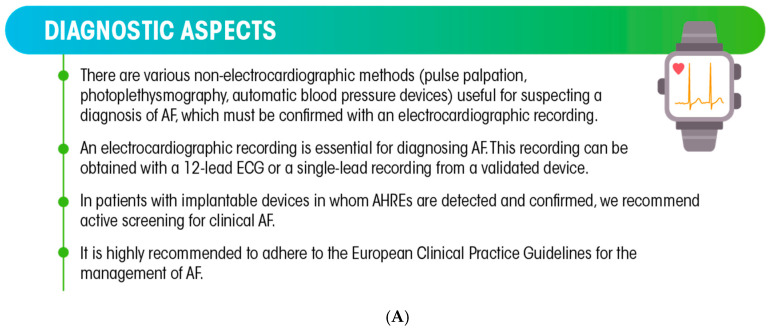

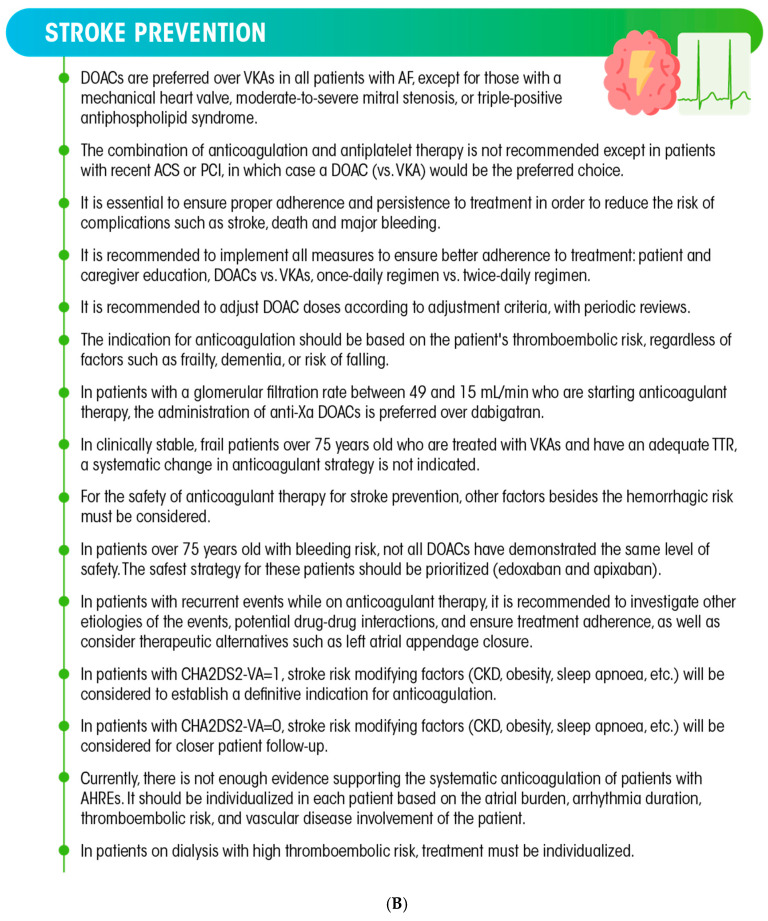

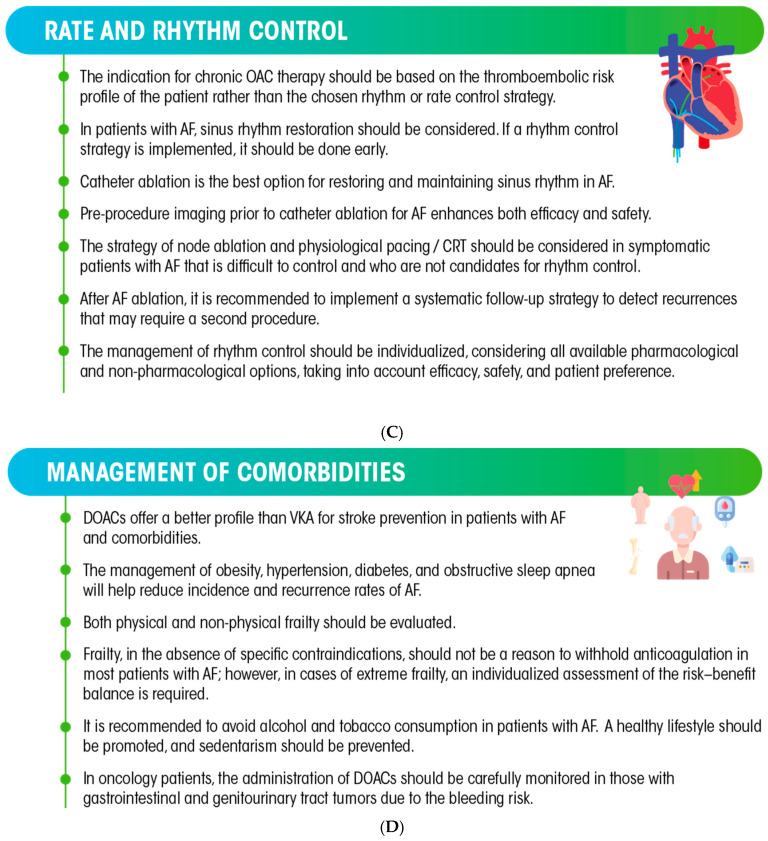
Practical recommendations for the comprehensive management of patients with AF. (**A**) Practical recommendations for diagnostic aspects; (**B**) Practical recommendations for stroke prevention; (**C**) Practical recommendations for rate and rhythm control; (**D**) Practical recommendations for management of comorbidities. ACS: acute coronary syndrome; AF: atrial fibrillation; AHRE: atrial high-rate episodes; CRT: cardiac resynchronization therapy; DOACs: direct oral anticoagulants; ECG: electrocardiogram; OAC: oral anticoagulant; PCI: percutaneous coronary intervention; TTR: time in therapeutic range; VKA: vitamin K antagonists.

The practical recommendations developed by the scientific committee and the panelists, based on the questionnaire results and the debate, are shown in Figure 1.

## 4. Discussion

Optimal management of AF remains a challenge, including diagnostic and anticoagulation strategies, patient adherence and persistence to treatment, optimization of rate and rhythm control, and the effective management of comorbidities and associated risk factors. Expert clinical judgment constitutes a valuable tool to address this challenge and support decision-making in an area where the existing evidence is not sufficient to cover the current gaps in knowledge. Therefore, this study provides practical recommendations based on expert opinions and relevant evidence to optimize the management of patients with AF.

### 4.1. Diagnostic Aspects

An early diagnosis of AF is crucial to prevent stroke and other consequent complications [13]. The updated European and American guidelines state that AF diagnosis should be confirmed by a specialist physician using an ECG [7,8], preferably a 12-lead ECG, and documenting episodes that last at least 30 s [7]. In accordance, experts recommend adhering to the European Clinical Practice Guidelines for the management of AF, and both the answers to the questionnaire and the practical recommendations highlight the importance of an electrocardiogram for diagnosing AF. This can be obtained with a single-lead recording from a validated device or, otherwise and preferably, with a 12-lead ECG.

The absence of symptoms makes diagnosis of AF difficult [7]. In this context, the use of wearable devices may help to diagnose subclinical AF, although confirmation by a competent professional reviewing the ECG recording is necessary [7]. Indeed, panelists disagreed (77% level of agreement) about recommending the use of validated wearable devices to assist in the diagnosis of subclinical AF. The high price of these devices and the difficulty for older patients to use them were highlighted as two important reasons for the discrepancy, and changing the statement to “In some cases, wearables are useful to detect AF” was proposed to achieve agreement. Nonetheless, experts recommend their use if there is a confirmation of AF diagnosis with an electrocardiographic recording. Several studies have shown reasonable performance of wearable devices in the diagnosis of AF [7,14]. The mSTOPS randomized clinical trial (RCT) shows that immediate monitoring with a home-based wearable ECG sensor patch resulted in a higher rate of AF diagnosis in individuals at high risk for AF [15]. Wearables such as the Apple Watch, AliveCor, or Huawei watches have proven to be useful in the detection of AF in various nonrandomized studies [14]. However, further RCTs and direct comparative studies are needed to ensure their effectiveness in clinical practice [7]. In any case, wearables should be validated to ensure the reliability of the data and their effective use in clinical practice [8].

Besides stroke prevention, AF screening has been shown to improve adherence to medication, risk factor control, and clinical outcomes [8]. Screening can be conducted systematically or opportunistically and can be performed at a single point in time or spread over a prolonged period. The optimal screening method will vary according to the population studied [7]. Guidelines state that AF screening is recommended in individuals over 75 years of age or those over 65 years of age with additional risk factors (CHA_2_DS_2_-VA score) [7]. This statement was agreed upon by the panelists with an 80% level of agreement. The remaining 20% level of disagreement could be due to the fact that it has not yet been established whether patients at high risk of developing AF benefit from screening interventions in terms of stroke and systemic embolism prevention or survival [8]. Additionally, in patients with implantable devices in whom atrial high-rate episodes (AHREs) are confirmed, experts recommend active screening for clinical AF.

### 4.2. Stroke Prevention

Atrial fibrillation is a major risk factor for stroke, especially when left untreated. Therefore, OACs should be provided to eligible patients, while antiplatelet drugs alone are not recommended for stroke prevention in AF [7]. Within OACs, it is well-known that DOACs are more effective than VKA in terms of stroke and major bleeding risk prevention [7,8,16,17], and thus, all panelists agreed with this statement (100% level of agreement). However, experts stated that DOACs are not the preferred option in patients with a mechanical heart valve, moderate-to-severe mitral valve stenosis, or triple-positive antiphospholipid syndrome, as the INVICTUS trial showed that, in patients with rheumatic heart disease and AF, VKAs provided more favorable outcomes compared to rivaroxaban [18,19,20]. The statement “Patients who may benefit from continued treatment with VKAs include those over 75 years of age, frail patients on VKA therapy who are clinically stable, and those for whom comorbidities and drug interactions contraindicate the use of DOAC” was met with only a 54% level of agreement. Panelists attributed the reason for the rejection to certain limitations of the FRAIL-AF study [21] and the conflicting nature of the statement, as experts generally agreed with the first part of the statement but not with the second. In any case, experts stated that clear and good communication and the patient’s opinion about the preferred therapy are crucial.

For patients with recurrent stroke, panelists disregarded the use of antiplatelet agents combined with anticoagulants, or the switch from one DOAC to another (63% level of agreement), as evidence concerning these topics is scarce and bleeding risk can potentially increase when administering combination therapy. Additionally, the dual nature of the statement may promote that panelists agree with one point but not with the other. Patients with acute coronary syndrome (ACS) or percutaneous coronary intervention (PCI) are the population in whom combined therapy (with DOAC instead of VKA) could be recommended. Experts also recommended evaluating adherence to the medication before modifying therapy, as switching to a once-daily DOAC may improve adherence and potentially outcomes.

Adherence and persistence to treatment are crucial aspects to consider in AF, as they are necessary to reduce the risk of complications [22]. Indeed, the lack of adherence and persistence to OACs is associated with a reduction in their efficacy and a worse prognosis [10]. For example, low adherence has been associated with a threefold increase in the risk of stroke, while low persistence has been linked to a twofold increase in this risk [23]. Generally, adherence to the medication decreases as the number of daily doses increases [10]. Therefore, once-daily DOAC, especially edoxaban, is associated with increased adherence and persistence [10,11,24], and panelists agreed with the corresponding statement. Experts also recommended prioritizing edoxaban and apixaban as the safest strategy in patients over 75 years old with increased bleeding risk, and using adherence aids such as pill boxes, reminders, or week organizers.

Direct oral anticoagulant dose adjustments emerged as another significant aspect to consider in AF. Recommended doses of DOACs are specified by different regulatory authorities [25]. Experts confirmed that incorrect dosing can increase thromboembolic risk and bleeding risk. Incorrect dosing should be avoided to optimize the efficacy and safety of DOACs [8,26]. Dose adjustment/reduction is required in some cases, such as in patients with low body weight, renal impairment, concomitant use of p-glycoprotein/CYP3A4 inhibitors, or the elderly, so an individualized treatment should be considered and periodically reviewed. According to the expert panel, some of the factors to consider for individualizing treatment include adherence to medication, concomitant medication, frailty, the presence of other comorbidities, and patient preferences. For example, in patients with chronic kidney disease (glomerular filtration rate between 49 and 15 mL/min), the anti-Xa DOACs are recommended over direct thrombin inhibitors, with edoxaban and apixaban being the preferred choices due to their capacity to cause significantly less major bleeding [27]. In line, the expert panel agreed with the statement “In patients with chronic kidney disease and creatinine clearance <50 mL/min, edoxaban and apixaban have demonstrated a significant reduction in major bleeding or severe bleeding” with a 94% level of agreement.

Catheter ablation has been shown to reduce AF burden [28]. A particularly controversial statement was whether OACs should be started three weeks prior to ablation in patients with low thromboembolic risk (31% level of agreement), although this was not the case for patients with high thromboembolic risk (94% level of agreement). Experts indicated that the reasons might be: (i) when the thromboembolic risk is very low (CHA_2_DS_2_-VA = 0), anticoagulation is not always essential, as there is no evidence which supports it; (ii) the guidelines do not specify anticoagulation in the case of low-risk patients; (iii) deciding whether to use anticoagulation also depends on the hemorrhagic risk; and (iv) the timing of anticoagulation may vary beyond the three-week window. Additionally, for patients undergoing ablation, panelists disagreed with systematic imaging to rule out thrombi before the procedure (71% level of agreement). Panelists stated that routine imaging for the detection of thrombi is not essential, but can be advisable, especially in patients with increased thrombotic risk, such as amyloidosis, hypertrophic cardiomyopathy, or rheumatic valvular disease. However, if proper anticoagulation has been administered during the three weeks prior to ablation, routine preprocedural TEE imaging is not obligatory, given the fact that imaging is performed during the ablation procedure.

### 4.3. Rate and Rhythm Control

The goals of long-term rhythm control are to maintain sinus rhythm, improve quality of life, slow the progression of AF, and potentially reduce morbidity related to AF episodes [7]. In this scenario, evidence shows that catheter ablation is superior to antiarrhythmic drugs in restoring and maintaining sinus rhythm [28]. Panelists agreed that catheter ablation is recommended over pharmacological treatment to maintain sinus rhythm. The choice of ablation technique and energy source may vary depending on the availability of resources at each center and the experience of the operators, and were not discussed in the present project. Nevertheless, it is worth highlighting that pulse field ablation is emerging as a cornerstone technique, offering a favorable safety profile and promising clinical outcomes [29,30]. In addition, the presence of AF triggers, such as supraventricular tachycardias, should be considered both when selecting candidates for ablation, and when determining the most appropriate procedural approach [31].

Remarkably, experts recommended pre-procedure imaging prior to catheter ablation to enhance both the efficacy and safety of the procedure, and a systematic follow-up strategy to detect recurrences that may require a second procedure. In any case, the management of rhythm control should be individualized, and all available pharmacological and non-pharmacological options and patient preferences should be considered.

Of note, panelists rejected two statements from this block. Firstly, “Pharmacological interactions between antiarrhythmic agents and anticoagulants are common and require rigorous monitoring” was met with a 57% level of agreement. Although it is known that pharmacological interactions between anticancer drugs, antiarrhythmic drugs, rate control drugs, and anticoagulants are common [8], experts pointed out that possible reasons for rejection were: (i) the meaning of “rigorous monitoring” is very broad and can lead to different interpretations or misunderstandings, and (ii) while it is possible and required to monitor patients on VKA, this is not routinely the case for patients on DOACs.

Secondly, “The duration of atrial high-rate episodes (AHREs) can guide the use of oral anticoagulation” showed only a 69% level of agreement. Although guidelines state that the duration of AHREs can guide oral anticoagulation (for example, OACs should be considered for patients with AHREs lasting ≥24 h) [8], there is still discussion about the specific management in case of shorter durations of AHREs. Indeed, in those episodes lasting between 6 min and 24 h, there is no clear evidence from RCTs—NOAH-AFNET 6 [32] and ARTESIA [33] trials—to support the use of DOACs. In those cases, panelists stated that AF burden (proportion of time (%) a person has high atrial rhythms and/or AF during a certain monitoring period), the presence of concomitant diseases, and the thromboembolic risk should be considered.

### 4.4. Management of Comorbidities

The presence of specific diseases or lifestyle habits might increase the risk of developing AF and may influence the choice of anticoagulant therapy [8,14]. The European guidelines on AF have given special attention to comorbidities both through the ABC pathways in previous editions and through the AF-CARE framework in the updated ones [7,34]. These strategies place the comprehensive care of concomitant diseases as the initial central component of the management of patients with AF [7]. In this regard, experts recommended that appropriately different concomitant diseases [such as obesity, hypertension, diabetes, and obstructive sleep apnea (OSA)] be managed to improve recurrence rates and the incidence of AF. Notably, in the case of OSA, treatment with continuous positive airway pressure has been shown to improve electrophysiological outcomes following AF ablation [35]. Overall, direct oral anticoagulants offer a better profile for stroke prevention in patients with AF and comorbidities.

Weight and body mass index (BMI) are important variables in drug distribution and plasma levels [26,36]. Panelists agreed that individuals with low body weight are at higher risk of overdose (and bleeding), while those with obesity are at higher risk of underdosing (and thromboembolic events), raising concerns about the efficacy and safety of DOACs at the extremes of weight [26,36]. In that context, experts recommended a healthy lifestyle and prevention of sedentarism in patients with AF. Furthermore, dose adjustments of edoxaban and apixaban are considered in low body weight individuals (≤60 kg), while a dose adjustment is not recommended for rivaroxaban or dabigatran [26].

Frailty is also associated with weight loss and deterioration of renal function, which implies close monitoring of body weight and renal function in these patients in order to adjust the dose of DOACs [26]. Specifically, it was recommended that both physical and non-physical frailty (e.g., cognitive, daily and social activities) be evaluated in patients with AF, and that frailty should not be a limiting criterion for anticoagulation. Only those frail AF patients with a clearly unfavorable benefit–risk balance, established after a careful comprehensive assessment, should be excluded from receiving anticoagulants [7].

Within the recommended healthy lifestyle habits, patients with AF were also encouraged to avoid alcohol and tobacco consumption, and panelists agreed with preferably recommending alcohol abstention rather than maintaining moderate consumption. This is crucial, as alcohol intake increases the risk of thromboembolism, death, or hospitalization in patients with AF and is associated with a higher risk of bleeding in patients on oral anticoagulants [7]. Additionally, in patients undergoing catheter ablation, alcohol consumption increases recurrence [7].

Patients with cancer generally present a higher risk of developing AF [7]. In this group of patients, DOACs present similar efficacy and a better safety profile compared to VKA [7], and thus, panelists agreed on the statement “In oncology patients with non-valvular AF and a favorable risk/benefit balance for anticoagulation, the prescription of DOAC is recommended”. However, experts also recommended careful monitoring of patients with gastrointestinal and genitourinary tract tumors, due to the increased bleeding risk.

### 4.5. Limitations of the Study

Despite the strengths of this methodology and the valuable recommendations generated through the anonymous questionnaire and in-depth face-to-face discussions, this study has certain limitations inherent to its design. First, it provides only qualitative information on the degree of agreement among panelists, based on relevant evidence, and their clinical practice experience and judgment. Second, due to the general scope of the work, and of both the literature review and the questionnaire, certain specific clinically relevant aspects, such as ablation techniques, the type of energy used, or triggers of paroxysmal AF, were not examined. The indications and technical considerations related to left atrial appendage occlusion, either as an alternative to antithrombotic therapy or in combination with this in cases of recurrent stroke, were also beyond the scope of this study and therefore not addressed. Finally, although the panel included highly experienced professionals with a strong publication record and representation across various regions of Spain, their selection was not systematic nor randomized.

## 5. Conclusions

Early diagnosis and management of AF is crucial to prevent the complications associated with this disease [13]. Electrocardiography (ECG), preferably a 12-lead ECG, is essential to diagnose AF [7]. This study emphasizes the use of wearable devices as a helpful tool to aid in the detection of AF, while confirmation by ECG is still necessary. The restoration and maintenance of sinus rhythm using catheter ablation is highlighted as a superior option compared to pharmacological treatment. Furthermore, the importance of managing any concomitant disease to improve recurrence rates and incidence of AF is emphasized. Patients with AF have increased risk of stroke, and therefore anticoagulant treatment should be provided to AF patients to prevent thrombosis and systemic embolism. In that context, this study highlights the benefits of DOACs over VKAs, the latter being preferred in patients with a mechanical heart valve, moderate-to-severe stenosis, or triple-positive antiphospholipid syndrome. Of all DOACs, both edoxaban and apixaban are the preferred choice in patients ≥75 years old and/or with renal impairment and increased bleeding risk. Once-daily DOACs have shown better adherence and persistence.

## Figures and Tables

**Table 1:** Sociodemographic characteristics of the participating panelists.

	Response	Number of Panelists *n* (%)
Specialty	Cardiology	33 (94%)
	Internal medicine	2 (6%)
Type of institution	Hospital	33 (94%)
	Outpatient clinic	2 (6%)
Working group member in a scientific society	Yes	23 (66%)
	No	12 (34%)
Geographical localization	Castile and León	2 (6%)
	Castile-La Mancha	1 (3%)
	Madrid	5 (14%)
	Andalusia	6 (17%)
	Valencian Community	5 (14%)
	Catalonia	5 (14%)
	Basque Country	2 (6%)
	Extremadura	1 (3%)
	Aragon	1 (3%)
	Galicia	3 (9%)
	Navarre	1 (3%)
	Canary Islands	1 (3%)
	Murcia	1 (3%)
	Cantabria	1 (3%)
Years of experience in the management of patients with AF and treatment with OACs	0–9	2 (6%)
	10–19	14 (40%)
	20–29	16 (46%)
	≥30	3 (8%)
Monthly number of patients with AF treated with OACs	25–50	17 (49%)
	≥50	18 (51%)

AF: atrial fibrillation, OACs: oral anticoagulants.

## Data Availability

The original contributions presented in this study are included in the article/Supplementary Material. Further inquiries can be directed to the corresponding author(s).

## Supplementary Materials

The following supporting information can be downloaded at: https://www.mdpi.com/article/10.3390/jcm14155199/s1, Table S1: Summary of eligibility criteria for publication inclusion; Table S2. List of selected publications included in the literature review; Table S3. Level of agreement of the different statements of the questionnaire.
