# Supplementary material for: Practical Comprehensive Approach to Current Atrial Fibrillation Challenges: Insights from an Expert Panel
Source: J Clin Med. 2025 Jul 22;14(15):5199. doi: 10.3390/jcm14155199 (PMC12346891; doi:10.3390/jcm14155199)
Supplement: Supplementary file 1 [file jcm-14-05199-s001.zip › jcm-3702302-supplementary.pdf]

Journal of
Clinical Medicine

MDPI

Supplementary Materials

Practical Comprehensive Approach to Current Atrial Fibrillation Challenges: Insights from an Expert Panel

Carlos Escobar [1,*], Miguel Camafort [2,3,4], Elena Fortuny [5], Maxim Grymonprez [6], Alejandro Isidoro Pérez-Cabeza [7,8], Tine L. de Backer [9] and Leaders Connect Group [†]

[1] Cardiology Service, University Hospital La Paz, 28046 Madrid, Spain
[2] Internal Medicine Service, Atrial Fibrillation Unit, Hospital Clínic Barcelona, 08036 Barcelona, Spain; camafort@clinic.cat
[3] Fundació de Recerca Clínic Barcelona-Institut d'Investigacions Biomèdiques August Pi i Sunyer (IDIBAPS), 08036 Barcelona, Spain
[4] Centro de Investigación Biomédica en Red Fisiopatología de la Obesidad y Nutrición (CIBER-OBN), 15706 Santiago de Compostela, Spain
[5] Cardiology Service, The Health Research Institute of the Balearic Islands (IdISBa), University Hospital Son Espases, 07120 Palma de Mallorca, Spain; elenafortunyfrau@gmail.com
[6] Pharmaceutical Care Unit, Department of Bioanalysis, Faculty of Pharmaceutical Sciences, Ghent University, 9000 Ghent, Belgium
[7] Cardiology Service, Clinic University Hospital Virgen de la Victoria, 29590 Málaga, Spain; alejandroipcabeza2@gmail.com
[8] Centro de Investigación en Red de Enfermedades Cardiovasculares (CIBERCV), 28029 Madrid, Spain
[9] Internal Medicine and Pediatrics, Department of Cardiology, Ghent University Hospital, 9000 Ghent, Belgium; tine.debacker@ugent.be
[10] Cardiology Service, Hospital de Manises, 46940 Valencia, Spain; julianabdala@gmail.com
[11] Cardiology Service, Hospital Universitario Fundación Jiménez Díaz, 28040 Madrid, Spain
[12] Medical School, Universidad Autónoma de Madrid, 28049 Madrid, Spain; alvaroacena@yahoo.es
[13] Hospital Universitario de Navarra, 31008 Pamplona, Spain; 37053oar@gmail.com
[14] Cardiology Service, Hospital Universitario La Paz, 28046 Madrid, Spain
[15] IdiPAZ Research Institute, 28029 Madrid, Spain; iantorrena@hotmail.com
[16] Hospital Universitario de Guadalajara, 19002 Guadalajara, Spain; jbalaguerr5@gmail.com
[17] Hospital Universitario Donostia, 20014 Donostia, Spain; jrberamendi@gmail.com (J.R.B.C.); irenerilo@gmail.com (I.RM.)
[18] Hospital Clínic Benidorm, 03501 Alicante, Spain
[19] Medical School, Universidad Miguel Hernández, 03202 Elche, Spain; vbertog@gmail.com
[20] Hospital Universitario Álvaro Cunqueiro, 36312 Vigo, Spain; cardiologiadrablanco@gmail.com
[21] Hospital Universitario Infanta Leonor, 28031 Madrid, Spain; semdoc@hotmail.com
[22] Cardiology Service, Arrythmia Unit, Hospital General Universitario de Elche, 03203 Alicante, Spain; jesus_kastillo@hotmail.com
[23] Internal Medicine Service, Hospital Clínico Universitario Lozano Blesa, 50009 Zaragoza, Spain; jcebollada@salud.aragon.es
[24] Cardiology Service, Hospital Universitario y Politécnico La Fe, 46026 Valencia, Spain; diadova@gmail.com
[25] Cardiology Service, Arrythmia Unit, Hospital Universitario Marqués de Valdecilla, 39008 Santander, Spain; vicast79@hotmail.com
[26] Cardidology Service, Electrophysiology and Arrhytmia Unit, Complejo Hospitalario Universitario de Canarias, 38320 San Cristóbal de La Laguna, Santa Cruz de Tenerife, Spain; juliojferrer@hotmail.com
[27] Cardiology Service, Complex Universitari Moisès Broggi, 08970 Barcelona, Spain; rfreixa@csi.cat
[28] Cardiology Service, Hospital de la Santa Creu i Sant Pau, 08025 Barcelona, Spain; xgarcia-moll@santpau.cat
[29] Cardiology Service, Hospital Universitario de Santiago de Compostela, 15706 Santiago de Compostela, Spain
[30] Cellular and Molecular Cardiology Unit, Institute of Biomedical Research of Santiago de Compostela (IDIS-SERGAS), 15706 Santiago de Compostela, Spain
[31] Centro de Investigación Biomédica Cardiovascular en Red (CIBERCV), Institute of Health Carlos III, 28029 Madrid, Spain; javiergarciaseara@yahoo.es (J.G.S.); jimenezcandil@secardiologia.es (J.J.C.)
[32] Cardiology Service, Hospital Universitario Torrecárdenas, 04009 Almería, Spain; cgnavarro@gmail.com
[33] F.E.A. Cardiology Imaging Unit, Cardiology Service, Hospital Universitario Virgen del Rocío, 41013 Sevilla, Spain; glezbarrero@hotmail.com
[34] Cardiology Service, Arrhythmia Section, Hospital General Universitario de Castellón, 12004 Valencia, Spain; claragunturizbeltran@gmail.com

Academic Editor: Gabriele Di Giammarco

Received: 30 May 2025
Revised: 2 July 2025
Accepted: 10 July 2025
Published: 22 July 2025

Citation: Escobar, C.; Camafort, M.; Fortuny, E.; Grymonprez, M.; Pérez Cabeza, A.I.; de Backer, T.L.; Leaders Connect Group. Practical Comprehensive Approach to Current Atrial Fibrillation Challenges: Insights from an Expert Panel. *J. Clin. Med.* **2025**, *14*, 5199. https://doi.org/10.3390/jcm14155199

Copyright: © 2025 by the authors. Licensee MDPI, Basel, Switzerland. This article is an open access article distributed under the terms and conditions of the Creative Commons Attribution (CC BY) license (https://creativecommons.org/licenses/by/4.0/).

[35] Cardiology Service, Hospital Universitario Rio Hortega, 47012 Valladolid, Spain; hgnacho@hotmail.com
[36] Servicio de Cardiología, Hospital Universitario de Salamanca. Instituto de Investigación Biomédica de Salamanca (IBSAL), 37007 Salamanca, Spain; jimenezcandil@secardiologia.es
[37] Departamento de Medicina, Universidad de Salamanca, 37007 Salamanca, Spain
[38] Hospital Universitario Clínico San Cecilio, 18007 Granada, Spain
[39] Instituto de Investigación Biosanitaria (IBS), 18012 Granada, Spain; jlozanoherrera62@gmail.com
[40] Hospital Central de la Defensa Gómez Ulla, 28047 Madrid, Spain
[41] Departamento de Medicina y Especialidades Médicas, Universidad de Alcalá, 28801 Madrid, Spain; docalcala@hotmail.com
[42] Cardiology Service, Hospital Juan Ramón Jiménez, 21005 Huelva, Spain; palomareu@gmail.com
[43] Hospital Universitario Vall d'Hebron, 08035 Barcelona, Spain; jfranciscopascual@gmail.com
[44] Cardiology Service, Hospital San Pedro de Alcántara, 10003 Cáceres, Spain; rosaporfer@yahoo.es
[45] Cardiology Service, Hospital Universitario Santa Tecla, 43003 Tarragona, Spain; serchmed7@hotmail.com
[46] Cardiology Department, Complexo Hospitalario Universitario de A Coruña (CHUAC), Sergas, 15006 A Coruña, Spain; jarofe@mundo-r.com
[47] Cardiology Service, Hospital Universitario Reina Sofía, 28702 Córdoba, Spain
[48] Maimónides Institute for Biomedical Research of Córdoba (IMIBIC), 14004 Córdoba, Spain
[49] Centro de Investigación Biomédica en Red de Enfermedades Cardiovasculares (CIBERCV), 28029 Madrid, Spain
[50] Medical School, Universidad Internacional Isabel I de Castilla, 09003 Burgos, Spain; maruor@gmail.com
[51] Hospital Universitario Puerta de Hierro, 28222 Madrid, Spain; adicerezo@gmail.com
[52] Cardiology Service, Arrhythmia Unit, Hospital Universitario Josep Trueta, 17007 Girona, Spain; metrucco.girona.ics@gencat.cat
[53] Internal Medicine Service, Hospital General Universitario Santa Lucía, 30202 Cartagena, Spain
[54] Department of Medicine, Universidad Católica San Antonio de Murcia, 30107 Murcia, Spain; javier.trujillosantos@gmail.com
[55] Cardiology Service, Hospital Universitario Costa del Sol, 29603 Marbella, Spain; almudenavalle@hotmail.com
* Correspondence: carlos.escobar@salud.madrid.org
† Julian Abdala-Lizarraga [10], Alvaro Aceña [11,12], Óscar Alcalde-Rodríguez [13], María Isabel Antorrena-Miranda [14,15], Javier Balaguer-Recena [16], Juan Ramón Beramendi-Calero [17], Vicente Bertomeu-González [18,19], Elisa Blanco-González [20], Sem Briongos-Figuero [21], Jesús Castillo-Castillo [22], Jesús Cebollada-del Hoyo [23], Diana Domingo-Valero [24], Victor Exposito-Garcia [25], Julio Jesús Ferrer-Hita [26], Román Freixa-Pamias [27], Xavier Garcia-Moll [28], Javier García-Seara [29,30,31], Carlos Gómez-Navarro [32], Antonio José González-Barrero [33], Clara Gunturiz-Beltrán [34], Ignacio Hernández-González [35], Javier Jiménez-Candil [8,36,37], José Miguel Lozano-Herrera [38,39], David Martí-Sanchez [40,41], Ana Blanca P. Martínez-Pérez [42], Jaume Francisco-Pascual [43], Rosa Porro-Fernández [44], Irene Rilo-Miranda [17], Sergio Rojas-Lievano [45], José Ángel Rodríguez-Fernández [46], Martín Ruiz-Ortiz [47,48,49,50], Adriana Saltijeral-Cerezo [51], María Emilce Trucco-Vernetti [52], Antonio Javier Trujillo-Santos [53,54] and Almudena Valle-Alberca [55].

Table S1. Summary of eligibility criteria for publication inclusion.

	Inclusion	Exclusion
Study type	Clinical practice guidelines<br>Systematic reviews and meta-analysis<br>Consensus documents<br>Randomized controlled studies<br>Observational studies	Ecological studies<br>Case series<br>Reports
Target population	Patients with AF	*In vitro* studies<br>Animal studies
Interventions	VKAs, DOAC, ablation, antiplatelet agents, rate-control and antiarrhythmic treatment	
Comparator	Placebo, no treatment or other pharmacological and non-pharmacological interventions	
Restrictions	Timeline: since 2014<br>Language: English or Spanish	Timeline: up to 2014<br>Language: others

AF: atrial fibrillation, DOAC: direct oral anticoagulant, VKA: vitamin K antagonist.

Table S2. List of selected publications included in the literature review.

PMID	Title	Year of publication	Journal
39210723	2024 ESC Guidelines for the management of atrial fibrillation developed in collaboration with the European Association for Cardio-Thoracic Surgery (EACTS)	2024	Eur Heart J
38033089	2023 ACC/AHA/ACCP/HRS Guideline for the Diagnosis and Management of Atrial Fibrillation: A Report of the American College of Cardiology/American Heart Association Joint Committee on Clinical Practice Guidelines	2023	Circulation
33895845	2021 European Heart Rhythm Association Practical Guide on the Use of Non-Vitamin K Antagonist Oral Anticoagulants in Patients with Atrial Fibrillation	2021	Europace
37952132	Apixaban for Stroke Prevention in Subclinical Atrial Fibrillation	2024	N Engl J Med
39143401	Effectiveness and safety of non-vitamin K antagonist oral anticoagulants in low-weight patients with atrial fibrillation	2024	J Thromb Thrombolysis
39288952	Management of atrial fibrillation in older adults	2024	BMJ
38291925	Practical guide on left atrial appendage closure for the non-implanting physician: an international consensus paper	2024	Europace
37634130	Safety of Switching From a Vitamin K Antagonist to a Non-Vitamin K Antagonist Oral Anticoagulant in Frail Older Patients With Atrial Fibrillation: Results of the FRAIL-AF Randomized Controlled Trial	2024	Circulation
37622677	Anticoagulation with Edoxaban in Patients with Atrial High-Rate Episodes	2023	N Engl J Med
37005194	Inappropriate prescriptions of direct oral anticoagulants (ACODs) in hospitalized patients: A narrative review	2023	Thromb Res
36769872	Use of Direct Acting Oral Anticoagulants in Elderly Patients with Atrial Fibrillation: A Multicenter, Cross-Sectional Study in Spain	2023	J Clin Med
36247477	Adherence and persistence to oral anticoagulants in patients with atrial fibrillation: A Belgian nationwide cohort study	2022	Front Cardiovasc Med
34780886	Comparison of clinical outcomes of edoxaban versus apixaban, dabigatran, rivaroxaban, and vitamin K antagonists in patients with atrial fibrillation in Germany: A real-world cohort study	2022	Int J Cardiol
35663451	Impact of undiagnosed obstructive sleep apnea on atrial fibrillation recurrence following catheter ablation (OSA-AF study)	2022	Int J Cardiol Heart Vasc
35790603	Increased Risk of Stroke Due to Non-adherence and Non-persistence with Direct Oral Anticoagulants (ACODs): Real-World Analyses Using a Nested Case-	2022	Drugs Real World Outcomes

	Control Study from The Netherlands, Italy and Germany		
35877831	Management of Atrial Fibrillation Across the Spectrum of Heart Failure With Preserved and Reduced Ejection Fraction	2022	Circulation
33554614	Ablation Versus Drug Therapy for Atrial Fibrillation in Heart Failure: Results From the CABANA Trial	2021	Circulation
33640355	Atrial Fibrillation Burden and Clinical Outcomes in Heart Failure: The CASTLE-AF Trial	2021	JACC Clin Electrophysiol
32920808	Edoxaban versus Warfarin in Patients with Atrial Fibrillation at the Extremes of Body Weight: An Analysis from the ENGAGE AF-TIMI 48 Trial	2021	Thromb Haemost
34141014	Electronic monitoring of adherence to once-daily and twice-daily direct oral anticoagulants in patients with atrial fibrillation: Baseline data from the SMAAP-AF trial	2021	J Arrhythm
34209595	Impact of Weight on Clinical Outcomes of Edoxaban Therapy in Atrial Fibrillation Patients Included in the ETNA-AF-Europe Registry	2021	J Clin Med
32324233	Long-term persistence and adherence with non-vitamin K oral anticoagulants in patients with atrial fibrillation and their associations with stroke risk	2021	Eur Heart J Cardiovasc Pharmacother
33357217	Clinical outcomes in patients with atrial fibrillation and frailty: insights from the ENGAGE AF-TIMI 48 trial	2020	BMC Med
32943160	Mortality in Patients With Atrial Fibrillation Receiving Nonrecommended Doses of Direct Oral Anticoagulants	2020	J Am Coll Cardiol
31753740	Vitamin K Antagonists and Direct Oral Anticoagulants in Nonagenarian Patients With Atrial Fibrillation	2020	J Am Med Dir Assoc
30691551	Concomitant Use of Direct Oral Anticoagulants with Antiplatelet Agents and the Risk of Major Bleeding in Patients with Nonvalvular Atrial Fibrillation	2019	Am J Med
31208303	Effectiveness and Safety of Contemporary Oral Anticoagulants Among Asians With Nonvalvular Atrial Fibrillation	2019	Stroke
30874766	Effect of Catheter Ablation vs Antiarrhythmic Drug Therapy on Mortality, Stroke, Bleeding, and Cardiac Arrest Among Patients With Atrial Fibrillation: The CABANA Randomized Clinical Trial	2019	JAMA
30612074	Non-vitamin K antagonist oral anticoagulants in elderly patients with atrial fibrillation: A systematic review with meta-analysis and trial sequential analysis	2019	Arch Gerontol Geriatr
31290171	Vitamin K antagonist vs direct oral anticoagulants with antiplatelet therapy in dual or triple therapy after percutaneous coronary intervention or acute coronary syndrome in atrial fibrillation: Meta-analysis of randomized controlled trials	2019	Clin Cardiol

26698882	Meta-Analysis of Renal Function on the Safety and Efficacy of Novel Oral Anticoagulants for Atrial Fibrillation	2016	Am J Cardiol
24315724	Comparison of the efficacy and safety of new oral anticoagulants with warfarin in patients with atrial fibrillation: a meta-analysis of randomised trials	2014	Lancet

Table S3. Level of agreement of the different statements of the questionnaire.

Topic	Statement	Agreement (%)	Level of agreement
AF diagnosis	In routine clinical practice, I follow the recommendations established in the updated national and international guidelines	100	Unanimity
	An electrocardiogram (ECG) is essential for diagnosing atrial fibrillation (AF)	89	Agreement
	In routine clinical practice, I recommend the use of validated wearables for patients to assist in the diagnosis of subclinical AF	77	Discrepancy
	Population screening for AF is justified in individuals aged 75 years or older, or those aged 65 years or older with additional risk factors (CHA_2DS_2-VA)	80	Agreement
Stroke prevention	Direct oral anticoagulants (DOAC) are preferred over vitamin K antagonists (VKAs) for the prevention of ischemic events and thromboembolic risk in patients with non-valvular AF	100	Unanimity
	DOAC are preferred over VKAs in patients with rheumatic valvulopathy without moderate or severe mitral stenosis	83	Agreement
	Patients who may benefit from continued treatment with VKAs include those over 75 years of age, frail patients on VKA therapy who are clinically stable, and those for whom comorbidities and drug interactions contraindicate the use of DOAC	54	Rejection
	In patients with recurrent stroke, the use of antiplatelet agents combined with anticoagulants, or switching from one DOAC to another, is not recommended	63	Rejection
	When combined antiplatelet and anticoagulant therapy is required, DOAC are preferred over VKAs	100	Unanimity
	In stroke prevention, appropriate dosing, adherence, and persistence with treatment are critical factors for the safety and efficacy of anticoagulation	100	Unanimity
	Poor adherence and/or poor persistence with anticoagulant treatment are associated with a higher risk of stroke	100	Unanimity
	A DOAC with once-daily dosing (edoxaban and rivaroxaban) is associated with higher adherence compared to twice-daily dosing (apixaban and dabigatran)	89	Agreement
	High-risk ischemic and hemorrhagic patients are more likely to receive incorrect DOAC doses, which increases their	91	Agreement

	thrombotic risk without reducing hemorrhagic risk		
	High-risk hemorrhagic patients do not benefit from subtherapeutic doses of DOAC	83	Agreement
	Among DOAC, edoxaban and apixaban have demonstrated a significant reduction in major bleeding or severe bleeding in patients over 75 years of age	100	Unanimity
	Falls, by themselves, do not constitute a contraindication for the use of DOAC	97	Agreement
	In patients with moderate to severe chronic kidney disease, with a creatinine clearance between 49 and 30 mL/min, I would recommend the administration of edoxaban, rivaroxaban, or apixaban instead of dabigatran	91	Agreement
	In patients with chronic kidney disease and creatinine clearance <50 mL/min, edoxaban and apixaban have demonstrated a significant reduction in major bleeding or severe bleeding	94	Agreement
	In patients with low thromboembolic risk, it is not necessary to initiate oral anticoagulation during the 3 weeks prior to ablation	31	Rejection
	In patients with high thromboembolic risk, imaging should be performed to rule out thrombi prior to an ablation procedure	71	Discrepancy
	Oral anticoagulation should be initiated at least 3 weeks prior to catheter ablation in patients with AF and high thromboembolic risk, and maintained for at least 2 months in all patients to prevent stroke	94	Agreement
Rate and rhythm control	Pharmacological interactions between antiarrhythmic agents and anticoagulants are common and require rigorous monitoring	57	Rejection
	The duration of atrial high-rate episodes (AHREs) can guide the use of oral anticoagulation	69	Discrepancy
	Catheter ablation improves survival, reduces the recurrence of AF, and enhances quality of life in patients with heart failure, compared to pharmacological treatment	94	Agreement
	Catheter ablation is more effective than antiarrhythmic drugs for restoring and maintaining sinus rhythm	94	Agreement
Management of comorbidities	Individuals with low body weight are at higher risk of overdose (and bleeding),	94	Agreement

	while those with obesity are at higher risk of underdosing (and thromboembolic events)		
	In diabetic patients, DOAC reduce vascular mortality compared to warfarin	89	Agreement
	In patients with AF and frailty, a comprehensive geriatric assessment is required to provide an adequate risk/benefit evaluation to make appropriate therapeutic decisions regarding stroke prevention and symptom control	91	Agreement
	Screening and proper treatment of sleep apnea syndrome in patients with AF should be prioritized before catheter ablation	91	Agreement
	In patients with AF, it is preferable to recommend abstaining from alcohol rather than maintaining moderate consumption	86	Agreement
	In oncology patients with non-valvular AF and a favorable risk/benefit balance for anticoagulation, the prescription of DOAC is recommended	97	Agreement
	Long-term anticoagulation has been shown to be beneficial in high thromboembolic risk patients with AF in the postoperative period following non-cardiac surgery	83	Agreement

AF: atrial fibrillation, AHREs: atrial high-rate episodes, DOAC: direct oral anticoagulant, ECG: electrocardiogram, VKA: vitamin K antagonist
